# “We thought we were stronger than we were”: adopters’ narratives about the adoption journey and disruption

**DOI:** 10.3389/fpsyg.2024.1381050

**Published:** 2024-04-08

**Authors:** Anca Bejenaru, Sergiu Raiu, Mihai Iovu, Alina Negoescu, Sorina Corman

**Affiliations:** ^1^Department of Social Work, Journalism, Public Relations, and Sociology, Faculty of Social Sciences and Humanities, Lucian Blaga University of Sibiu, Sibiu, Romania; ^2^Department of Human and Socio-Political Sciences, Faculty of History and Geography, Stefan cel Mare University of Suceava, Suceava, Romania; ^3^Social Work Department, Faculty of Sociology and Social Work, Babes-Bolyai University of Cluj-Napoca, Cluj-Napoca, Romania; ^4^Department of Applied Social Sciences and Humanities, Faculty of Military Sciences, Nicolae Bălcescu Land Forces Academy, Sibiu, Romania

**Keywords:** adoption disruption, adopters’ experiences, special needs adoption, adoption support, psychological and social services

## Abstract

**Introduction:**

Although the majority of adoptive families remain stable, some of them break up prematurely.

**Methods:**

Adopting a qualitative approach, this study gave voice to seven adoption applicants who began the adoption journey with one or more children but did not complete the legal process. Our goal was to understand their experiences throughout the adoption process and disruption.

**Results:**

The results show how adoption applicants went, in a short period, from the excitement of finding a child or sibling group to disillusionment and trauma. The main risk factors that hinder adoption stability include unrealistic expectations, intuitive choice of the child in the absence of accurate information, challenges posed by the child’s particular characteristics, and lack of professional support.

**Discussion:**

Our study highlights the need for adopters to be assisted throughout the entire process by specialists, to be better prepared to deal with the complex needs of children in the protection system, and to facilitate their access to a complex of specialized services to meet the different needs of every family.

## Introduction

Disruption is one of the terms most often used in the literature to denominate the failure of the adoption process for children whose biological parents cannot look after them. However, its use in the studies is inconsistent (Palacios et al., 2019; Lyttle et al., 2021). For instance, in England, the concept describes the child’s separation from the potential adopters before and after legal completion (Selwyn et al., 2014; Lyttle et al., 2021). In the United States, the term is used narrowly to denote the termination of adoption before legal finalization (Rosenthal, 1993), while the term dissolution was preferred for denominating post-legal adoption separation.

In Romania, adoptions can be disrupted only during entrustment, a three-month period before the adoption is legally completed.^1^ This period may be extended under exceptional conditions up to 6 months. During this period, the child lives with the prospective adoptive parents, and adoption specialists monitor their adaptation. After the legal completion, the adoption can be terminated only after the child turns 18, under exceptional circumstances.^2^

### Romanian legal and social context on child adoption and disruption

In Romania, until 2016, adoption was practiced exclusively based on matching procedures established recently by a national electronic system, including persons certified for adoption and adoptable children. In the matching process, the system considers children’s characteristics and the adoption criteria set by the certified persons. As most Romanians set as criteria the youngest age, good health, and no disabilities (Buzducea and Lazăr, 2011), children who did not fit these characteristics had no real chance of being adopted domestically. Intercountry adoption is not an option either. Through a moratorium instituted in 2001 and later through the Adoption Law of 2004, the adoption of children by foreign citizens was prohibited. This ban was caused by the large number of international adoptions registered after the fall of the communist regime in 1989, favored by the less regulated procedures and by the concerns caused by the limited capacity of Romanian bodies to monitor children adopted abroad (Popescu et al., 2020). In the period that followed, 2001–2016, Romania made no effort to stimulate national adoptions (Bejenaru, 2017).

Since 2016, a series of measures have been taken, both by simplifying the procedures and reducing the time for declaring adoptable children, as well as by adopting some measures to stimulate the adoption of hard-to-adopt children (HtAC). One such measure was creating a list of children with a public profile, initially called the list of hard-to-adopt children. Children for whom a family is not found within 6 months or for whom matching fails are registered on this list. This list can be viewed by all persons regardless of the number and profile of the children for whom they were initially certified, and they can choose to initiate the adoption process with any child/sibling group on the list without benefiting from additional training. In this way, children with health problems, disabilities, older children, and sibling groups have an extra chance to be adopted.^3^ Academic literature includes these categories of children under the generic term children with special needs (Rosenthal, 1993; Child Welfare Information Gateway, 2010; Pecora, 2010; O’Dell et al., 2015), or the more contested term hard-to-place children. Henceforth, we will use both the terms children with special needs and HtAC children as appropriate. Since 2016, HtAC adoptions in Romania have increased annually. Thus, while in 2016, there were two HtAC adoptions, in 2021, their number reached 260. During this period, an increase has also been registered among the children adopted through matching, from 1,053 registered in 2016 to 1,393 in 2021 (NAPRCA, 2022). In addition to these adoptions that were successfully completed, several adoptions were disrupted during the entrustment period, both from among the children adopted through matching and from among the HtAC. Statistically, HtAC adoptions have a higher disruption rate than matched adoptions. From the data provided in 2022 by 40 of the 47 public institutions responsible for adoption in Romania, we found that in the period 2016–2021, the average rate of disrupted adoptions for matching adoptions was 1.9%, while the average rate of disrupted adoptions for children chosen from the HtAC was 13%.

In the next section, we will review the literature to highlight the main factors that compete for disruption and how adopters experience this process.

### Factors affecting the stability of adoption of children with special needs

#### Adopters’ profiles and the motivation to adopt a child with special needs

While in Romania, the adoption of children with special needs has only recently started to be encouraged through policies and practices, in other countries, such as the US or the UK, it has been promoted for over five decades (Carp, 2002). The first studies, which attempted to capture adopters’ profiles and motivations, have been available since the 1970s. These studies showed that the characteristics of adopters of older children, those with health problems and disabilities, or siblings distinguish them from the majority. According to the findings of Deiner et al. (1988), many of these children were adopted from foster families with above-average incomes and strong religious beliefs. The families were cohesive, adaptable, and flexible. Their motivation was mainly to provide stability and security for the child and less to improve their health problems. Similarly, McRoy (1999) showed in her studies that in stable adoptions, adopters have a strong marriage, and are flexible and mature. They prove commitment to adoption, have experience in raising children, have a vast support network, and their expectations of the child are realistic. In some cases, they have cared for children as foster parents. Adopters’ motivation is altruistic rather than centered on personal fulfillment (McRoy, 1999). Support for the importance of this motivation and commitment to children to ensure adoption stability comes from more recent studies (Denby et al., 2011; Paniagua et al., 2022). According to Paniagua et al. (2022), motivations centered on adult desires for parenthood and companionship present a higher risk of disruption. Not all adopters enter the adoption process intending to adopt children with special needs. An interesting classification is proposed by Lindstrom et al. (2013), in which parents are divided into three categories: those who, from the outset, wish to adopt a child with special needs, those who initially wish to adopt a healthy child and then decide that they can accept for adoption a child with special needs, and those who adopt an apparently healthy child but later discover that he or she has special needs.

#### Preparing adopters to meet the challenges posed by the child’s characteristics

The literature is mainly convergent, with more significant risks of instability and disruption for children with special needs (McDonald et al., 2001). However, “special needs” is an umbrella term that covers children with very different characteristics. Among these, behavioral and emotional disorders and difficulties in forming attachment bonds with prospective adopters seem to be the most predisposing factors for adoption failure (Selwyn et al., 2014; Palacios et al., 2019).

The risk of disruption also increases with the child’s age. The more time the child spends in the care system, the more trauma and developmental delays accumulate that affect their perception of self and others, self-control ability, and behaviors (Palacios et al., 2019; Lanham, 2022). As for the presence of disabilities and medical problems, as long as they are known and assumed by the parents before the adoption, they seem to put less at risk the stability of the adoption. Moreover, Mozzi and Nuernberg (2016) showed that a disability may contribute to the intensification of emotional bonds between adoptees and adopters because of the child’s dependence on the adopters and more significant requests for care.

Adopters appear to be generally less prepared not only when faced with the behavioral disorders but also with relationship and attachment difficulties exhibited by children (Valentine et al., 1988; Wind et al., 2005; Tonheim and Iversen, 2019). Some of them only understand the extent of these problems after placing children in their own families (McRoy, 1999). Prior studies show that adopters need more practical training and a concrete understanding of their challenges after placement (Bergsund et al., 2018; Rosenthal et al., 1996; Rushton and Monck, 2009). According to other studies, adoptive parents often claim that they receive incomplete information about their children (Reilly and Platz, 2003; Coakley and Berrick, 2007; Lee et al., 2018) or that specialists present the children’s situation in a way that is quite favorable to increase their chances of adoption (Selwyn et al., 2014), generating unrealistic expectations in adopters, that may predispose to disruption (Coakley and Berrick, 2007). Other studies believe pre-adoption training has not helped them develop the skills to cope with the challenges they face after placement (Rosenthal et al., 1996; Rushton and Monck, 2009).

#### Experience with adoption specialists and support services

There is a consensus in the literature concerning the prevention of disruptions, particularly in the adoption of children with special needs. This involves identifying issues as early as possible and providing appropriate support (Haugaard et al., 2000). Lack of or fragmented provision of support services predisposes to disruption (Barth and Miller, 2000). During this short “trial” period, contact with adoption services specialists seems extremely important for adopters. Schmidt et al. (1988) showed that adoptive parents valued specialists’ honesty, openness, and empathy. Their support helped parents maintain respect and confidence in their ability to cope with difficult situations and readjust. The same study showed the frustration of adopters who were not believed by specialists when they told them about the severity of their children’s behavioral disorders. Similarly, in the study conducted by Lyttle et al. (2021), many parents claimed that they felt judged, unheard, or not trusted. Another study conducted in the U.S. showed that 88% of adopters who experienced disruption felt that lack of emotional support from adoption professionals and lack of appropriate response/communication from adoption services were significant barriers to successful adoption (USDHHS, 2020).

### Experiences throughout the adoption and disruption process

In earlier studies, parents argued about the isolation and alienation during entrustment (Valentine et al., 1988). Some claimed that they did not find understanding and support from the outside, and their feelings were not validated (O’Neill, 1993; Bergeron and Pennington, 2013). The process of disruption was perceived as traumatic by many of them (McKirdy et al., 2019). According to Schmidt et al. (1988), the adopters experienced the loss of the child following disruption, like the loss of a person through death. These results are supported by a recent study conducted by Goldberg and Allen (2022) on LGBTQ adopters. In the study led by O’Neill (1993), some parents reported anger and regret, while others felt shame or guilt. These and other feelings, such as stress, grief, despair, exhaustion, and relief, were also identified in the research conducted by Lyttle et al. (2021).

Although prior studies yielded significant data on the factors that affect adoption stability, only some studies have focused on the entrustment stage, although most adoptions fail before their legal completion (Selwyn et al., 2014). Also, very few studies have addressed the manner in which adopters experience the adoption journey and its disruption during the entrustment stage. Therefore, our study aims to fill this gap.

### Research objectives

The goal of this study was to understand the participants` experiences throughout the adoption and disruption process and the challenges they encountered in their journey with the child or siblings entrusted for adoption.

Specifically, our objectives sought to:

(1) explore the motivations to adopt a child from the HtAC list; (2) analyze how they appreciated their preparedness for the process; (3) identify the challenges the children’s characteristics posed and their ability to cope with them; (4) describe the experience with adoption specialists and support services, and (5) to capture how they felt about the adoption journey and disruption process.

## Materials and methods

The present study is part of a larger research project that focused on the resilience of the adoptive family in Romania. To capture resilience, within this project, we triangulated the ecosystem perspective and that of family development, as proposed by Walsh (2007). The ecosystemic perspective allowed us to study resilience as a multilevel interaction process between the adoptive family and other complex or challenging systems (Ungar, 2015), while the developmental perspective facilitated the understanding of adverse factors over time and gave coherence to the study.

The research project included both adopters who completed the process and currently have a stable adoption, as well as participants who interrupted this process. In the current study, only the latter were included.

### The profile of participants

Of the 41 participants in the study, seven met the following inclusion criteria: they had initiated the adoption procedure for a child/sibling group from the list of HtAC during 2018–2021, and they requested the revocation of the adoption before its legal completion. Six of the selected participants were female, and one participant was male. Among them, one adopter was unmarried, and six were married. The participants were approached individually, but in the case of a couple, both partners wanted to participate in the study and were accepted. Their responses were processed and reported separately. The parents’ ages ranged from 37 to 51, with a mean age of 41. All the participants in the study have completed higher education and have well-developed careers in different fields: two work in educational services, two in public administration, two offer professional and technical services, and one in financial services. The study refers to nine children. Four were placed alone, and the others in sibling groups of two and three. The children spent between four and 20 weeks with the adoptive family. Table 1 contains more details about the children and study participants.

**Table 1:** Socio-demographic data on adopters and entrusted children.

	Data about adopters				Data about the entrusted child				
Cod*	Gender	Age (years)	Education	Marital status	Age (years)	Gender	Adopted alone / with siblings	Health status/ disabilities	Entrustment length (weeks)
Tania	female	40	higher education	married	8	female	alone	hyperkinetic disorder, dyslexia	4
Marta	female	41	higher education	married	5	female	sibling group	language delays	4
					3	female		healthy	
					4	male		hearing deficit	
Adriana	female	51	higher education	married	10	female	alone	healthy	20
Dan Bianca	male female	40 42	higher education	married	6	female	sibling group	healthy	20
					10	female		healthy	
Oana	female	36	higher education	single	6	female	alone	healthy	8
Carmen	female	37	higher education	married	8	female	alone	healthy	4

*The names of the participants have been changed to ensure confidentiality.

### Data collection and processing

For this study, a qualitative methodology based on a semi-structured interview was adopted. The interview guide was developed based on the theoretical perspectives that substantiated the research. Some of the topics were: motivation for adoption, choosing the child from the list of HtA children, issues in the adoption process, social services needed and available, the disruption decision, and adopters’ thoughts and feelings throughout the process. Data collection was carried out between August 2022 and March 2023.

The interviews were conducted by two members of the research team, the principal investigator, specialized in psychology, with expertise in adoption, and a researcher, specialized in sociology, with expertise in investigating different categories of vulnerable people.

Because of the great geographical disparity of research participants, the interviews were conducted by telephone and audio recorded. Although this interview technique allows less access to non-verbal data, previous studies have proven that the telephone interview can provide rich narrative data about sensitive topics (Padilla et al., 2022), is less intrusive, and gives participants more power to control the interview situation (Drabble et al., 2016). After they expressed their interest in participating in the study, they were contacted and further informed about the purpose of the research, the estimated duration of the interview, the way of processing and storing the collected data, and the lack of any economic advantages resulting from their participation. This information was messaged to participants, along with the consent form. The participants established the time coordinates for the interview to fit their schedules and minimize the risk of withdrawal. The interviews lasted between 42 and 68 min, averaging 56 min. All of them were verbatim transcribed by the interviewers. The interviewing strategy closely followed the recommendations made by Glogowska et al. (2011) and Drabble et al. (2016) and was considered appropriate for our study as well. This study was approved by the Committee on Ethics in Scientific Research Involving Human Subjects of Lucian Blaga University, approval no. 7 on July 29, 2022.

### Data analyses

The data were processed using NVivo12 qualitative data analysis software. They were analyzed thematically by two of the research team members, closely following the six phases of the analysis process proposed by Braun and Clarke (2006, 2019).

The first phase involved familiarization with the data by reading and re-reading them repeatedly. The two researchers coded the data independently in the second phase, adopting an open-coded procedure. However, from the beginning, there was a consensus among researchers to follow the factors that triggered the transition to different stages in the adoption process (e.g., initiating the process, giving up matching, choosing the child from the list, and disrupting the process), but also the challenges that the participants faced in different stages of this process. This way, we ensured we would code relevant information to answer the research questions. A similar technique was used by Byrne (2022). In the third phase, the coders reviewed and discussed the individually generated codes. In this phase, a series of codes were collapsed, and those considered irrelevant to the research questions were eliminated. By grouping the remaining codes and analyzing the links between them, nine themes were developed in the fourth phase. and later defined in the fifth phase. After completing this stage, in order to facilitate the understanding of the adoption journey, followed by the disruption, we chose to group the themes and present them according to the phases of the adoption process, as follows:

The journey of adoption until entrustment with the themes:

- the decision-making process to adopt,
- giving up the matching process,
- intuitive choice in the absence of accurate information,
- failure to identify the potential risk factors for adoption success.

The entrustment period and disruption with the themes:

- challenges caused by the child’s characteristics,
- the adopters’ feelings during entrustment,
- the experiences of adoptive parents with professionals and support services.

After disruption with the themes:

- parents’ thoughts and feelings,
- thinking of new adoption.

In the sixth phase, all authors contributed to writing and reviewing the research results to ensure they provided a meaningful picture of the participants’ narratives.

## Results

### The journey of adoption until entrustment

#### The decision-making process to adopt

Two sub-themes emerged in the respondents’ narratives about the decision to adopt: the reasons behind the decision and how they reached it, which we will discuss further.

##### Reasons for adoption

In the case of all study participants, the decision to adopt was based mainly on unfulfilled personal wishes. The most frequently mentioned desire was to have a child, which could not previously be satisfied for various reasons. Therefore, four couples talked about infertility or health problems that prevented women from carrying a pregnancy to term. For example, Marta (F, 41) stated: *“We have infertility problems and after a few years, because we want children, we decided to resort to adoption.”* Another respondent who wanted a child was unmarried, while a couple whose partners were 40 and 44 years old at the time of the decision to have a child thought it was too late to try naturally.

Other desires mentioned by an applicant for adoption who had a biological child were to expand her family and also to have a healthy child. She explained as follows: *“We wanted to expand our family. We have another child with disabilities, and we wanted one more, healthy child.”* (Carmen, F, 37).

The reasons focused on the adopters’ desires were doubled by philanthropic reasons, focused on the children’s needs, in the case of the unmarried person, and those who considered that they were too old to procreate. The desire to help a child was evident from their narratives. For example, Oana (F, 36), unmarried, stated: *“The desire to give a disadvantaged child a few more chances for harmonious and continuous development.”* and Tania (F, 40), married, said: *“We thought that if there were still 60,000 or more children in the care system, we could undoubtedly do better for one of them.”*

##### How the adoption decision was reached

The respondents also told in their narratives how they came to the adoption decision. For the unmarried person, but also for the couple where the partners thought that their age was too advanced to have a child naturally, adoption represented the only option to satisfy their desire to have a child. For example, Tania (F, 40), married for 24 years, during which time she dedicated herself to her professional career, stated:

*"We spent our youth working, and then we realized we were relatively old and had accumulated many resources. After excluding having a biological child (…), we decided to adopt".*

For others, adoption was the second-best option or even the last. One couple decided to adopt after a period of *“unsuccessful attempts to have a biological child “*(Marta, F, 41). In contrast, other couples tried various methods of assisted reproduction, turning to adoption as a last resort. For example, Bianca (F, 42) mentioned: *“We tried to have a baby, and after three unsuccessful IVFs, this was the final solution.”*

### Giving up the matching process

Once the decision of adoption was made, most adopters wanted a small child without significant health problems. No family was explicitly motivated to adopt an older child, with disabilities, health problems, or behavioral disorders. Most of the children on the list do not meet their expectations, and yet most choose to view these lists and begin adoption proceedings for a child/sibling group on it. Adopters report being discouraged by the long waiting time for a matching with a child who meets the initial criteria, and encouraged by social workers, choose to view the list to shorten this process. Others feel they have reached an age where they can no longer wait. For example, Adriana (F, 51) said:

"*I obtained that certificate, and after I was told that I had to wait a long time for the matching and since my age did not allow me to wait for many years, I said that I would go to the list of hard-to-place children.*"

In some cases, adopters went to the list of HtAC with unrealistic expectations induced by the specialists who trained them for adoption. To get as many potential adopters as possible to view the list and to improve the chance of children being adopted, some experts unrealistically describe the list. In this sense, Marta (F, 41) stated:

"*We went to the list of hard-to-adopt children because that was how we're educated in training. We're told to focus on the older kids; the kids on the list, that it's wonderful (…) that it's ideal to go for an older child because they already understand adoption.*"

### Intuitive choice in the absence of accurate information

Adopters’ choice from the list of HtAC is often intuitive, without fully understanding the diagnoses and having very accurate information available about children. For example, Bianca (F, 42) stated: “*We saw pictures and read the data. We tried to understand the profile descriptions, although we did not understand many diagnoses.*” The same adopter reported that she chose two sisters from the list, of different ages, but their descriptions were the same. She continued: “*The synthetic sheet was wrong because it was copy-pasted from the first to the second, meaning both sisters had the same information, even the date of birth, even though it was not so. (…) They both had the same characteristics presented there.*” Other adopters have reported that the children’s photos were not updated. The information about the children’s diagnoses is not accurate either, and most parents assert that they “*cannot rely on what is written there*” (Adriana, F, 51). The diagnoses were more severe for some children, while they were not confirmed for others. For example, Oana (F, 36) stated: “*The child was sociable, playful, intelligent (…). There were various diagnoses, which were not confirmed during the subsequent checks.*”

Since the children on the HtAC list can be from any Romanian county, the specialists in charge of the adopters do not know any additional data about the children, apart from the ones registered in the system. From some of the participants’ narratives, it appeared that the professionals who were responsible for the children did not know them very well either. For example, Tania (F, 40) said: *“They [the specialists] had not seen the child for about two years. They were shocked at how much she had grown.”*

### Failure to identify the potential risk factors for adoption success

Reflecting on the period before entrustment, most adopters thought at that time that the happiness of finding the child/children and the enthusiasm of the immediate placement made them less receptive to specific indications that could have represented alarm signs for the stability of the adoption and less objective in analyzing the situation. Therefore, the majority declared that they had no doubts at the beginning about the success of the adoption. Tania (F, 40) said:

"*But we had already passed the stages and were very happy… So, I refused to listen and understand what I heard then.*"

Similarly, Marta (F, 41) recounted:

"*When you have wanted children for a long time, and you get to know them, and like that, I don't know, objectivity disappears, I'm telling you honestly. Looking back, I judge things differently, but we were excited then; we wanted it to happen and.*"

A single interviewee who opted to adopt a 10-year-old girl declared that she had doubts about the adoption’s success. These doubts concerned the desire of the adopter to “model” the adopted child and the genetic inheritance of some mental health problems that were present in the biological mother. She said:

"*A little, yes, because of the age; when I realized that she was so old, I didn’t think that… I mean, it was a risk anyway, not being able to transform her too much. But after that, I said let's start this little adventure anyway. I mean, you don't have to have prejudices. I started with the idea that we should not have prejudices, that even if her biological mother had schizophrenia, she did not necessarily have to develop schizophrenia; I consulted specialists, and they told me to try anyway…*" (Adriana, F, 51)

### The entrustment period and disruption

#### Challenges caused by the child’s characteristics

Most entrusted children were registered as physically healthy, and the rest with minor health problems. Two had language delays, and one had hearing impairments. These deficiencies were presented in the children’s files, and the adopters did not consider them impediments to the adoption’s success. Instead, children’s behavior and psychological states posed considerable challenges for which adopters were unprepared. They talked about the psychological disorders manifested by some of the children, which took the form of aggression toward the adoptive family members, self-aggression, and even suicidal tendencies. In some cases, these behaviors were exhibited by children when they had minor frustrations, as Adriana (F, 51) reported: *“So, she had a lot of neurotic attacks. She was screaming, kicking her feet, and banging her head against the walls, for whatever reason (…) like if someone said to her, for example, you are acting like a baby, she was acting badly.”* Similarly, Carmen (F, 37) narrated: *“Oppositional, aggressive attitudes: she tore sheets of paper, personal items, damaged furniture when limits were imposed on her. The limits were respecting the daily routine: changing from pyjamas to daywear clothes and doing homework.”* In other cases, it was out of a desire to interrupt an activity or to get what they wanted. Tania (F, 40) talked about the risky behaviors of the girl entrusted to her because of the child desire to be taken back to foster care: *“There were moments when she wanted to throw herself off the balcony if they did not take her back. If I walked with her on the street, she would jerk my hand and tell me she would throw herself in front of the car if I did not take her back.”* The same adopter argued that the girl grabbed her neck and put the jacket over her eyes while driving the car to avoid taking her to the dentist.

Other children did not have aggressive behaviors but showed hyperactivity and other behaviors that the adopters managed with difficulty. Marta (F, 41) said that one of the three entrusted children was taken for a walk every morning, at 5 o’clock, by car so that the other children could sleep. In this case, the specialists consulted by adopters after entrustment suspected several related mental illnesses: ADHD, autism spectrum disorder, and fetal alcohol syndrome, which the adopters did not know about before placement.

Most adopters declared that they had created weak attachment bonds with children during the time spent in the family. In three cases, the children had a powerful bond with the foster caregivers who raised them from a very young age. Oana (F, 36) declared that “*Taking the child from the family where he grew up is too brutal. The child had a strong bond with the former caregivers, making adapting to another entirely different family difficult*.” Similarly, Adriana (F, 51) stated, about the 10-year-old girl who was entrusted to her: *“That girl had been raised by a foster caregiver since she was seven months old. Do you realize she was like a mother to her? When my husband and I showed up, the girl was already very attached to this foster caregiver.”* In another case, the entrusted girl kept in touch with the biological family through the foster parents without being legally allowed, after the child was declared adoptable. Therefore, in her situation, there was a breakdown in her relations with both the foster and birth families. In these cases, adopters talk about children’s lack or poor preparation for adoption.

Another issue raised by respondents was the lack of any progress in the children’s development during the entrustment period. Despite the efforts of the adopters and professionals called upon, several children exhibited deficits in cognitive and language development and did not make any improvement. Dan (M, 40) remembered:

*"After they came to our house, we slowly realized that the older one did not speak at all, not even a ‘yes’ or a ‘no’. It was tough. Come on, a day, two, three, a week, but she didn't speak for three months. And for us, it was something atypical. So no, we couldn't imagine… it was like working with a child with Alzheimer. So, every day, we started over and over again, and nothing changed. On the contrary, it was worse".*

#### The adopters’ feelings during entrustment

The period of entrustment, although awaited by the adopters, was felt by the majority as highly stressful, full of uncertainties, and seeking specialized services and solutions for children’s issues. Some of the adopters felt significantly emotionally affected during this period. Dan (M, 40) declared that he “*had a nervous breakdown”* after 3 months of entrustment and Bianca (F, 42) stated that she started having “*panic attacks*.” Another adopter felt that this period created a lot of imbalances in the couple. She stated: “*I was scared, panicked, and realized that I could not live with a psychologically unbalanced person in the house because this would also unbalance my husband and me, and we were about to get a divorce*” (Adriana, F, 51).

#### The experiences of adopters with professionals and support services

From the adopters’ statements, some benefited from the necessary support from the public services that mediated the entrustment. However, they said nothing could have been done for the stability of the adoption. In this regard, Marta (F, 41) argued: *“The psychologist and the social worker came about once a week. (…) So, the psychologist was very OK. So, she came and talked to us.”* Similarly, Oana (F, 36) related, *“There was support throughout the whole process, as much as necessary.”* Other adopters did not request support, believing they did not need it because *“it was too late”* (Carmen, F, 37). They believed the children should have been better prepared for adoption, which would have prevented disruption.

However, most adopters asked for support and did not receive it. Some were directed to seek private, professional services on their own. Other specialists transferred the responsibility of the intervention from one public institution to another. Tania (F, 40) presented their situation like this:

*"We tried to talk to the Directorates, and every three days, we talked to everyone again. What should we do? Help! Help us! The child is crying, she wants to go to her foster care. Help! Help! They sent us from one to another. Everyone said that the girls were no longer their responsibility. So, I didn't get support…"*

Most adopters claimed that they did not find support even while they were in the process of disruption. They felt misunderstood, blamed, and, in some circumstances, compelled to maintain the child or sibling group. For instance, Carmen (F, 37), referring to specialists from the public directorate, stated:

*"The revocation of the adoption was accompanied by abuses and attempts to persuade us to keep the kid even if we realized that we were incompatible."*

Adopters claimed that specialists were mainly concerned with the children and disinterested in the mental health of other adoptive family members. Bianca (F, 42) stated:

*"I argued with the lady from the Directorate on the phone. I told her that we were not psychologically OK. And she told me: ‘I'm not interested in how you are; I'm interested in how the children are.’ So, I felt so bad, and that lady told me we were mocking children, and so on. That really hurt me."*

### After disruption

#### Adopters’ thoughts and feelings

Some adopters have described the whole experience of entrustment and then revocation as painful and even traumatic. A particular situation was presented by Marta (F, 41), who had three children entrusted. The family would have liked to keep two of them, with whom they managed to create an emotional bond during the entrustment period, but felt they could not cope with the special needs of the third. Although the revocation occurred 2 years ago, Marta (F, 41) stated that they have not fully recovered:

*"I suffered a lot after that period; I still can't say I'm completely over it. The emotional impact is powerful. It was a period that I tried to get over, but you can't get over it. I keep thinking about them, I keep thinking about how they are, if they're okay, if… You can’t forget. It's been two years and…"*

#### Thinking of new adoption

After the revocation, one of the families managed, to successfully adopt two girls. Another two families have relinquished their certificate and, therefore, their right to adopt. The rest of the families have kept their certificates and are considering adopting. Most are reluctant to see the list of children with public profiles again and wait for a match with a child based on their criteria. Marta (F, 41) visited the list after the revocation of the first adoption, but she did not choose any child, showing more caution. None of the families received psychological counseling post-revocation, but the specialists advised all of them to take a break before trying a new adoption.

## Discussions

Our study gives voice to seven adoption applicants who have begun the adoption journey with one or more children without the process being legally completed. Our goal was to understand their experiences throughout the adoption process and disruption. They recounted how they went, in a short time, from the hope of becoming adopters to the excitement and happiness of finding a child or sibling group and then to disillusionment, despair, and trauma. Several risk factors that can hinder adoption success can be drawn from their stories. Their understanding can inform social policy and practice to prevent similar situations.

### Giving up theoretical matching and false expectations

Matching in child adoption is an old procedure, and empirical evidence shows that some of its components significantly predict adoption stability (Lanham, 2022). According to Farmer and Dance (2016), *matching* can be defined as fitting adopters’ strengths or resources with the child’s or sibling group’s needs. In Romania, as in most legislative systems, the matching process involves two phases: theoretical matching, based on recorded data about the child or the group of siblings and applicants for adoption, and practical matching, when the two parties meet. All our respondents have begun their adoption journey with the desire to adopt children as young as possible (most under the age of three) and without significant health problems or disabilities. They are those adopters who primarily wanted to parent a child, and the motivation to help a child was secondary and directly expressed only in two cases. Most chose adoption as the second or even the last option to become parents. Giving up the theoretical matching process and choosing a child from the HtAC list meant several concessions from the adopters, mainly related to age and, in some cases, the number of children. There are several reasons why adopters give up theoretical matching. Adoption workers warn adopters from the beginning that this procedure takes a long time because there are no children available according to their initial criteria. This practice is also highlighted by other studies (Burge and Jamieson, 2009). A second reason for abandoning matching is that specialists encourage adopters to consider the HtAC list and focus on older children. The participants report that the list was presented to them much too favorably. The specialists aim to increase the chances of adopting as many children as possible, especially when there is a policy to encourage adoptions among children with special needs. These practices create unrealistic expectations for adopters, and according to previous studies, they are highly prone to disruption (Barth and Miller, 2000; Coakley and Berrick, 2007). Furthermore, prior research indicates that not all parents are suitable to adopt older children or children with special needs (Brind, 2008). However, according to the Romanian adoption procedure, any certified person or couple can choose a child from the HtAC list, regardless of their profile, without requiring additional training.

### Intuitive choice and lack of accurate information

The choice of the child or group of siblings on the HtAC list was based more on the adopters’ intuition. They talk about the lack of assistance viewing the HtAC list, not understanding the children’s diagnoses, and the incomplete or inaccurate information recorded about the child or children. They report that even the specialists responsible for adoptable children do not know the children’s situation closely. In many studies, the lack of adequate information and preparation of adopters to address the requirements of older children, with behavioral disorders, and sometimes with disabilities, is associated with instability or adoption disruption (Rosenthal, 1993; Reilly and Platz, 2003; Santos-Nunes et al., 2018).

### Adopters’ failure to understand and to meet and fulfill the needs of the children

Deficiencies in informing and preparing adopters lead to their failure to identify the children’s profile and needs accurately. Children’s characteristics *per se* do not predispose to disrupted adoptions, but rather the adopters’ false expectations and lack of adequate preparation and resources to deal with the children’s needs. Except for one participant, all others stated that they had no doubts about the success of the adoption before entrusting the child or sibling group.

Some failed to understand the potential attachment difficulties of children. They expected to give and receive love from their child or sibling group. It is well known, from previous studies (Howe, 2001; van den Dries et al., 2009; White, 2016) that children with a history of trauma present adaptive survival strategies, which do not allow them to initially perceive the adopters as sources of care and protection (Howe, 2001), thus it takes a long time to bond and offer affection. For some adopters, the apparent indifference of the child/children and attachment difficulties soon became unacceptable and created dissatisfaction. These results are consistent with those reported by Wydra and O’Brien (2018), who demonstrated that family cohesion and adoption satisfaction are predicted by parental ratings of affective responsiveness. According to previous studies, children adopted at older ages may show indifference or rejection to the adopters’ initial efforts to create an emotional bond with them (Quinton et al., 1998). However, they argue that parents must be adequately prepared to expect such rejections and not give up, as in time the affection will become mutual. Other studies have also shown that newer adoptive parents believe that adopted children show a willingness to form new attachments and that a secure and protective environment is enough to ensure the adaptation of the children (Ward, 1997; Santos-Nunes et al., 2018). According to some of our interviewees, children entrusted to them were very attached to the foster caregivers in whose care they had been for a long time.

Other adopters expected to see the rapid recovery of the child’s cognitive and language delays amidst specialized support and a stable environment and felt discouraged when they found that recovery was very slow or even imperceptible. In their study, Moyer and Goldberg (2017) showed that when adopters find that they have little power to shape children, they can feel a great deal of frustration. Among the participants who adopted sibling groups, some realized that cumulative costs of the children’s treatment and recovery needs exceeded the family’s resources.

For half of the participants, the most serious issues they faced were children’s behavioral problems and their lack of skills to deal with them. These results agree with earlier research, which shows that behavioral disorders exhibited by children seem to have the most significant influence on adoption stability (Rosenthal and Groze, 1990; Smith and Howard, 1991; McGlone et al., 2002; Nalavany et al., 2009).

Facing all these challenges from children, some adopters felt emotionally affected, exhausted, and overwhelmed, others argued that their marital relationship had been destabilized, and others feared for themselves or other family members. Similar experiences were also reported by Lyttle et al. (2021), in their research.

### Lack of support

One participant tried to end the adoption without seeking specialist help believing it would be better for the child to return to the foster family to whom they had become attached. In two other cases, the adopters felt they had received as much help as needed but without positive results. The remaining respondents requested support but did not receive it during the entrustment period or the disruption procedure. Some respondents perceived that specialists were exclusively focused on the children’s welfare, neglecting the adopters’ well-being. For this reason, the respondents felt they were not listened to, misunderstood, and even ignored. When they reached the point of requesting disruption, some felt compelled to retain the child or sibling group despite being accused and blamed. Adopters in other research expressed similar feelings (Lyttle et al., 2021). None of the study participants received psychological counseling post-disruption. The adoption specialists recommended only taking a break before considering another adoption. Many people argue about the trauma that persists long after the disruption occurs, even though the children have spent relatively short periods with the family. These findings are consistent with those of Goldberg and Allen (2022), who studied 80 LGBTQ people who went through different experiences of loss related to adoption.

### Implications

The results of this study can be used to shape social policies and practices, reducing adoption instability and disruption. The opening of the HtAC list is a procedure that offers an additional chance for adoption for many children for whom a suitable family could not be found in a short time. While most adoptions from this list are stable, some disrupt soon after entrustment. The unrestricted access to adoption from this list for all certified persons could be problematic because it may lead to unstable adoptions for at least two reasons. Firstly, most prospective adopters must give up their initial preferences, which may leave them feeling unfulfilled. Secondly, they may not possess sufficient resources and preparation for adopting a group of siblings or a child with behavioral, health, or disability problems. Thus, it is essential to ensure that abandoning initial preferences for adoption and selecting a child from the list is accompanied by additional training sessions and psychological counseling. This will allow adopters to enter the process with conviction, fully comprehending the needs of these children and committed to their welfare and upbringing.

The selection of a child from the HtAC list should be guided by specialists who can clarify the child’s diagnostics and explain the potential challenges posed by the child’s age, social history, health status, and potential disabilities. Adoption specialists must ensure that they know the children closely and that the information provided about the children is up-to-date and accurate. Before fostering, specialists should ensure that prospective adopters spend enough time with the child in different environments to get to know him or her as well as possible and that the child is sufficiently prepared for separation from the foster carers.

For adoption to be stable, all parties involved need to feel well. Therefore, it is, imperative that in the monitoring process, not only the children be assessed and supported but also the parents. Counseling and parenting skills development services that meet the unique needs of adopted children, crisis intervention, and even respite centers must be accessible to prospective adopters at all times.

### Limitations

The disruption problem has yet to be studied in the Romanian context, and it is not a visible issue in the public discourse on child adoption. Nevertheless, a low number of adopters are eager to share their unsuccessful adoption stories indicating potential trauma and the fear of being misunderstood or blamed for the failure (O’Neill, 1993). As a consequence, for our study, we were able to identify a total of seven adopters from six families who had attempted to adopt a total of nine children. Three participants were referred to us by adoption workers, and others were identified through social networks, thus reducing the potential bias that might have occurred if all adopters were identified using a single strategy. However, the results cannot be generalized because of the small number of participants. Another limitation of the study is that it solely reflects the views of the adoption applicants and does not consider the perspective of the adoption workers and entrusted children. More comprehensive studies are required to better understand the experiences of all parties and the risk factors that lead to disruption to prevent as many of these situations as possible.

## Data availability statement

The raw data supporting the conclusions of this article will be made available by the authors, without undue reservation.

## Ethics statement

The studies involving humans were approved by Committee on Ethics in Scientific Research Involving Human Subjects of Lucian Blaga University, approval no. 7 on July 29, 2022. The studies were conducted in accordance with the local legislation and institutional requirements. The participants provided their written informed consent to participate in this study.

## Author contributions

AB: Writing – original draft, Writing – review & editing. SR: Writing – original draft, Writing – review & editing. MI: Writing – original draft, Writing – review & editing. AN: Writing – original draft, Writing – review & editing. SC: Writing – original draft, Writing – review & editing.

## Footnotes

^1^Given this aspect, for the present study, we will use the restricted meaning of the term disruption to refer to the separation of the child from the prospective adopters during entrustment.

^2^See Romanian Civil Code of July 17, 2009, Section 4. Adoption termination, Article 477 (3).

^3^The procedure for adopting children from the list of children with a public profile is provided in Decision no. 798 of July 28, 2021, issued by the Government of Romania.
